# Atrial Fibrillation Related and Unrelated Stroke Recurrence Among Ischemic Stroke Patients With Atrial Fibrillation

**DOI:** 10.3389/fneur.2021.744607

**Published:** 2021-10-21

**Authors:** Bum Joon Kim, Yang-Ha Hwang, Man-Seok Park, Joon-Tae Kim, Kang-Ho Choi, Jin-Man Jung, Sungwook Yu, Chi Kyung Kim, Kyungmi Oh, Tae-Jin Song, Yong-Jae Kim, Kwang-Yeol Park, Jeong-Min Kim, Jong-Ho Park, Jay Chol Choi, Jong-Won Chung, Oh Young Bang, Gyeong-Moon Kim, Sung Hyuk Heo, Woo-Keun Seo

**Affiliations:** ^1^Department of Neurology, Asan Medical Center, Seoul, South Korea; ^2^Department of Neurology, Kyungpook National University Hospital, Daegu, South Korea; ^3^Department of Neurology, Chonnam National University Hospital, Gwangju, South Korea; ^4^Department of Neurology, Korea University Ansan Hospital, Ansan, South Korea; ^5^Department of Neurology, Korea University Anam Hospital, Seoul, South Korea; ^6^Department of Neurology, Korea University Guro Hospital, Seoul, South Korea; ^7^Department of Neurology, Ewha Womans University Seoul Hospital, Seoul, South Korea; ^8^Department of Neurology, Eunpyeong St. Mary's Hospital, The Catholic University of Korea, Seoul, South Korea; ^9^Department of Neurology, Chung-Ang University Hospital, Seoul, South Korea; ^10^Department of Neurology, Seoul National University Hospital, Seoul, South Korea; ^11^Department of Neurology, Myongji Hospital, Hanyang University College of Medicine, Goyang, South Korea; ^12^Department of Neurology, Jeju National University Hospital, Jeju, South Korea; ^13^Department of Neurology, Samsung Medical Center, Seoul, South Korea; ^14^Department of Neurology, Kyung Hee University Hospital, Seoul, South Korea

**Keywords:** atrial fibrillation, cardioembolic brain infarction, recurrence, stroke, stroke mechanism

## Abstract

**Background:** Ischemic stroke with atrial fibrillation (AF) may recur despite appropriate treatment. It may be AF-related or AF-unrelated. We compared the factors associated with AF-related and AF-unrelated recurrences among ischemic stroke patients with AF.

**Methods:** Patients with ischemic stroke and AF were enrolled from 11 centers in Korea. Ischemic stroke recurrence was classified as AF-related if the lesion pattern was compatible with cardioembolism without significant stenosis or as AF-unrelated if the lesion was more likely due to small vessel disease or arterial stenosis. Factors associated with stroke recurrence (AF-related and AF-unrelated) were investigated.

**Results:** Among the 2,239 patients, 115 (5.1%) experienced recurrence (75 AF-related and 40 AF-unrelated). Factors independently associated with any stroke recurrence included AF diagnosed before stroke, small subcortical infarctions, and small scattered lesions in a single vascular territory. Type of AF was associated with the type of stroke recurrence, with persistent AF being associated with AF-related stroke [hazard ratio (HR) = 2.94, 95% confidence interval (CI) 1.69–5.26; *p* < 0.001]. By contrast, paroxysmal AF (HR = 3.76, 95% CI 1.56–9.04; *p* = 0.003), AF diagnosed before stroke (HR = 2.38, 95% CI 1.19–4.55; *p* = 0.014), small scattered lesions in a single vascular territory (reference: corticosubcortical lesion, HR = 3.19, 95% CI 1.18–8.63; *p* = 0.022), and the use of antiplatelet agents (HR = 2.11, 95% CI 1.11–4.03; *p* = 0.024) were independently associated with AF-unrelated stroke.

**Conclusion:** Persistent AF was more associated with AF-related stroke recurrence, whereas paroxysmal AF was more associated with AF-unrelated stroke recurrence. A scattered lesion in a single vascular territory may predict AF-unrelated stroke recurrence.

## Introduction

Despite being appropriately anticoagulated, a considerable proportion of ischemic stroke patients with atrial fibrillation (AF) experience recurrence (1). Factors [p]predictive of recurrence in patients with cardioembolic stroke (CE) include older age, presence of diabetes, large lesion, and left atrial enlargement (2). Another factor that may be associated with recurrence is the mechanism of stroke; more specifically, AF may be an innocent bystander, with alternative mechanisms precipitating ischemic stroke (2). A quarter of CE patients have concomitant high-grade carotid stenosis (3), whereas 10–15% of ischemic stroke patients with AF present with lacunar infarction. Approximately one-third of patients with AF seem to have cerebral infarction not associated with the cardioembolic source (AF-unrelated ischemic stroke) (4).

Predicting the risk of AF-related and AF-unrelated stroke may be important to determine the secondary prevention strategy. The CHA2DS2-VASC score is widely used to determine the risk of CE (5). However, the components of the CHA2DS2-VASC score are also risk factors for atherosclerosis and small vessel disease, both of which may increase the risk of AF-unrelated stroke recurrence (6). Therefore, there have been efforts to focus on the cardiac source itself to predict true AF-related stroke recurrence; previously, findings of echocardiography were related with the type of stroke recurrence (7). The characteristics of AF itself are also associated with the risk of stroke recurrence. The type of AF (persistent vs. paroxysmal AF) and its time of diagnosis (before vs. after stroke), which are associated with stroke recurrence, may also influence the type of stroke recurrence among ischemic stroke patients with AF (8). As ischemic stroke lesion patterns represent the stroke mechanism, it may also influence the type of stroke recurrence (9). The present study comprehensively investigated and compared the clinical and radiological factors associated with AF-related and AF-unrelated recurrences among ischemic stroke patients with AF.

## Materials and Methods

### Participants

This was a sub-study of the Korean ATrial fibrillaTion EvaluatioN regisTry in Ischemic strOke patieNts (K-ATTENTION) study. The K-ATTENTION study database prospectively enrolled ischemic stroke patients with AF evaluated at 11 centers in South Korea from January 2013 to December 2015. The collection of data has been described (10), with a total of 3,213 patients enrolled. Clinical data were obtained from a retrospective review of medical records. All patients underwent standard evaluation. Patients also received standard acute stroke treatment care according to the protocol of each participating center. Patients were excluded if (1) cerebrovascular imaging data were unavailable, (2) clinical data were incomplete, or (3) follow-up data were unavailable. The study protocol was approved by the ethics committee of each participating institution, which waived the requirement for written informed consent due to the retrospective design. The institutional review board (IRB) number of the affiliated center of the first author was 2016-08-039.

### Clinical Laboratory and Imaging Data

Electrocardiograms were obtained from the emergency department and were monitored during patient admission to stroke units. Transthoracic echocardiography was performed. Additional Holter monitoring was performed in patients who were suspected to have an embolic source. Based on these results, paroxysmal AF was defined as AF that terminated within 7 days of onset, and persistent/permanent AF was defined as AF that failed to self-terminate within 7 days or during the admission. The time of AF diagnosis was categorized as before or after the index stroke. Further extensive investigation including transesophageal echocardiography, cancer workups, or laboratory tests for coagulopathy were performed based on local protocols. The use of antithrombotics was determined by attending vascular neurologist at each center. Usually, patients with active bleeding, low platelet count, systemic coagulopathy, or large infarction with high risk of hemorrhagic transformation or those refusing anticoagulation did not receive any antithrombotics or antiplatelet agents, based on the physicians' decision.

All patients underwent diffusion-weighted imaging (DWI) and magnetic resonance angiography (MRA) within 24 h of admission. Lesion patterns and the presence of atherosclerosis were investigated. All imaging data were interpreted by experienced neurologists and neuroradiologists at each center who were blinded to patients' clinical factors. DWI lesion pattern was centrally reviewed by additional investigators (BK and W-KS).

### Recurrence of Stroke

Recurrence of ischemic stroke between January 2013 and May 2017 was determined at each study center by investigators during regular or unscheduled visits during follow-up. Recurrent stroke was defined as a clinically evident stroke with a newly developed neurological deficit and a corresponding lesion confirmed by neuroimaging. All patients received magnetic resonance image (MRI) including DWI to determine the recurrent stroke mechanism, and all medical records of each center were reviewed by an independent investigator (W-KS) to confirm a recurrent event.

Recurrent AF-related ischemic stroke was defined as a stroke with an ischemic lesion compatible with CE without significant stenosis at the corresponding artery. Recurrence of AF-unrelated ischemic stroke was defined as (1) an isolated small (maximum diameter <1.5 cm) ischemic lesion at the location of the perforating artery territory or (2) a single or multiple scattered lesions restricted to a single artery territory with corresponding significant stenosis at the proximal artery. The type of recurrent stroke was categorized by local investigators and was confirmed during centralized analysis by two experienced neurologists with consensus meetings (BK and W-KS).

### Statistical Analysis

Clinical and radiological factors were compared between patients with and without ischemic stroke recurrence and between patients with AF-related and AF-unrelated stroke recurrence. Pearson's chi-square test, Fisher's exact test, and Student's *t*-test were appropriately used. The occurrence of AF-related and AF-unrelated stroke according to the time from index stroke was presented in those with paroxysmal and persistent AF. Factors associated with AF-related and AF-unrelated stroke were investigated by univariable and multivariable analyses using binary logistic regression. To avoid variable selection caused by spurious correlations, only variables showing a potential association (*p* < 0.1) in univariable analysis were included as potential factors associated with stroke recurrence in the multivariable logistic regression model. Statistical significance was defined as a two-tailed *p* < 0.05. All statistical analyses were performed using SPSS 21.0 (IBM Corporation, Armonk, NY, USA).

## Results

**Figure 1:**
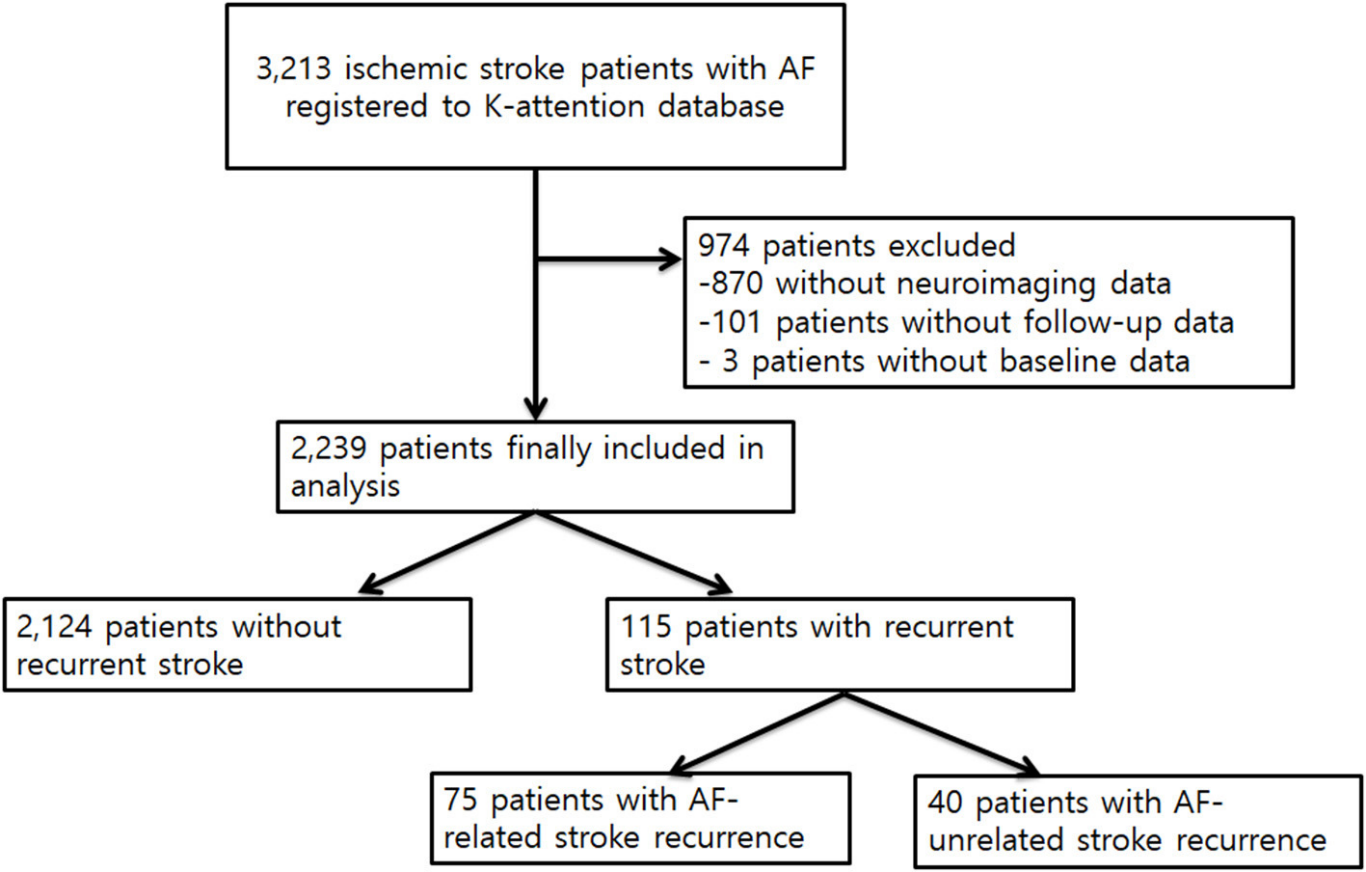
Study profile.

Of the 3,213 patients enrolled in the K-ATTENTION study, 974 were excluded, including 101 patients without follow-up data, 870 patients without neuroimaging data, and three patients with incomplete workup. Thus, 2,239 patients were enrolled in this analysis (Figure 1). The mean follow-up duration was 1.46 years.

Of the 2,339 enrolled patients, 115 (5.1%) experienced recurrence of ischemic stroke. Risk factors did not differ significantly between patients with and without ischemic stroke recurrence, except that the prevalence of dyslipidemia was significantly higher in patients with stroke recurrence. The percentage diagnosed with AF before the index stroke was higher in patients with ischemic stroke recurrence (Table 1). Multivariable analysis showed that recurrence of any stroke was independently associated with AF diagnosed before the index stroke [hazard ratio (HR) = 1.82, 95% confidence interval (CI) 1.23–2.7; *p* = 0.002] and lesion patterns of subcortical infarction (HR = 2.38, 95% CI 1.24–4.59; *p* = 0.009) and small scattered lesions in a single vascular territory (HR = 2.00, 95% CI 1.10–3.64; *p* = 0.024).

**Table 1:** Baseline characteristics of study patients.

	**No recurrence** **(*n* = 2,124)**	**Any recurrence** **(*n* = 115)**	***p*-value**	**AF-related** **(*n* = 75)**	**AF-unrelated** **(*n* = 40)**	***p*-value**
Age (years)	73.5 ± 10.0	73.0 ± 9.1	0.609	73.7 ± 9.7	71.7 ± 7.9	0.252
Male sex	1,099 (51.7)	50 (43.5)	0.084	32 (42.7)	18 (45.0)	0.810
Hypertension	1,446 (68.1)	80 (69.6)	0.739	53 (70.7)	27 (67.5)	0.725
Diabetes	540 (25.4)	37 (32.2)	0.107	27 (36)	10 (25.0)	0.229
Dyslipidemia	424 (20.0)	33 (28.7)	0.024	24 (32)	9 (22.5)	0.283
CAD	285 (13.4)	21 (18.3)	0.141	16 (21.3)	5 (12.5)	0.243
Smoking	286 (13.5)	14 (12.2)	0.692	11 (14.7)	3 (7.5)	0.263
CHF	85 (4.0)	6 (5.2)	0.520	5 (6.7)	1 (2.5)	0.339
CHA_2_DS_2_-VASC score	5 (4–6)	5 (4–6)	0.052	6 (4–6)	5 (4–6)	0.078
Type of AF			0.140			<0.001
Persistent	977 (46.0)	61 (53.0)		55 (73.3)	6 (15.0)	
Paroxysmal	1,147 (54.0)	54 (47.0)		20 (26.7)	34 (85.0)	
AF diagnosis			0.002			0.289
Before index stroke	1,081 (50.9)	76 (66.1)		47 (62.7)	29 (72.5)	
After index stroke	1,043 (49.1)	39 (33.9)		28 (37.3)	11 (27.5)	
Lesion pattern			0.226			0.367
Corticosubcortical	569 (26.8)	19 (16.5)		12 (16.0)	7 (17.5)	
Cortical	204 (9.6)	13 (11.3)		8 (10.7)	5 (12.5)	
Subcortical > 15 mm	165 (7.8)	9 (7.8)		7 (9.3)	2 (5.0)	
Subcortical <15 mm	115 (5.4)	6 (5.2)		3 (4.0)	3 (7.5)	
Small scattered	220 (10.4)	18 (15.7)		9 (12.0)	9 (22.5)	
Confluent + additional	429 (20.2)	27 (23.5)		22 (29.3)	5 (12.5)	
Multiple vascular territory	422 (19.9)	23 (20.0)		14 (18.7)	9 (22.5)	
Carotid stenosis	560 (26.4)	22 (19.1)	0.085	13 (17.3)	9 (22.5)	0.502
Mechanism of stroke			0.376			0.300
CE only	1,781 (83.9)	100 (87.0)		67 (89.3)	33 (82.5)	
Two or more	343 (16.1)	15 (13.0)		8 (10.7)	7 (17.5)	
Medications at discharge			0.227			0.103
Anticoagulants	1,574 (74.1)	79 (68.7)		56 (74.7)	23 (57.5)	
Antiplatelet agents	451 (21.2)	32 (27.8)		16 (21.3)	16 (40.0)	
None	99 (4.7)	4 (3.5)		3 (4.0)	1 (2.5)	
Follow-up duration	1.48 (0.15–2.57)	1.09 (0.2–1.87)	<0.001	1.17 (0.16–2.26)	0.95 (0.24–1.59)	<0.001

*Results are presented as number (% column), mean ± SD, or median (IQR). P-values were calculated using the chi-square test or Student's t-test.*

*AF, atrial fibrillation; CAD, coronary artery disease; CHF, congestive heart failure; CE, cardioembolism*.

**Figure 2:**
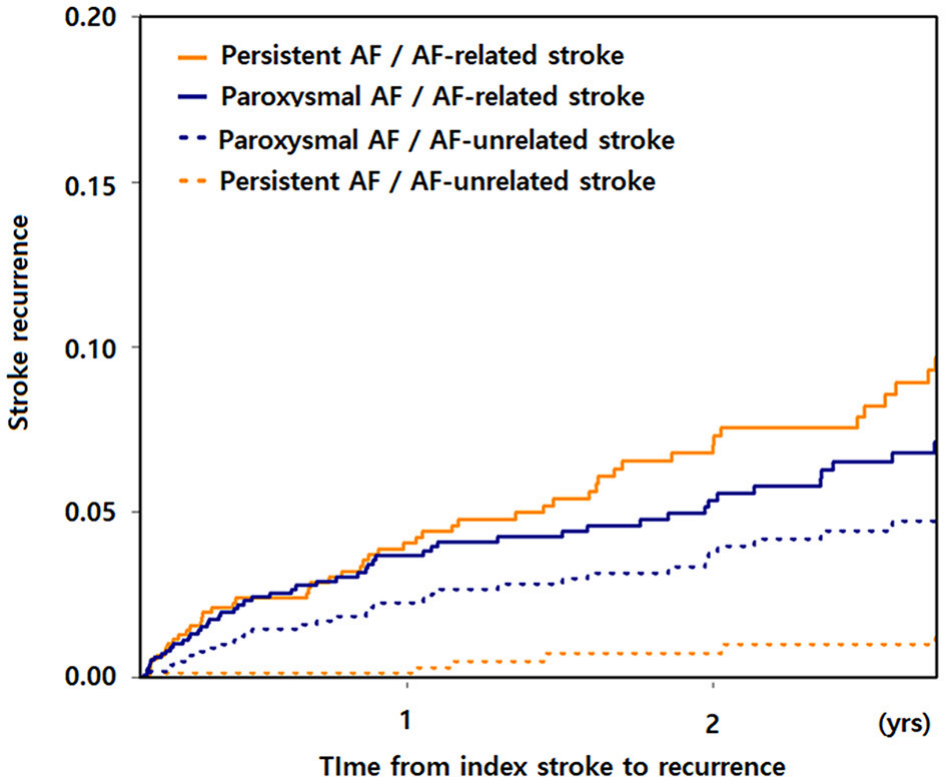
AF-related and AF-unrelated stroke recurrence from index stroke according to the type of AF. AF, atrial fibrillation.

Of the 115 patients with ischemic stroke recurrence, 75 (65.2%) had AF-related and 40 (34.8%) had AF-unrelated ischemic stroke recurrence. Although risk factors did not differ significantly between these two groups, the type of AF differed: the prevalence of persistent AF being higher in patients with AF-related stroke and the prevalence of paroxysmal AF being higher in patients with AF-unrelated stroke (Table 1). Regarding the initial lesion pattern, corticosubcortical infarction pattern was the most common in patients without stroke recurrence. In comparison, the pattern of confluent lesions with additional small lesions in a single vascular territory was the most common in patients with AF-related stroke recurrence, and the pattern of small scattered lesions in a single vascular territory was the most common in patients with AF-unrelated ischemic stroke recurrence. The recurrences of AF-related and AF-unrelated stroke after the index stroke according to the type of AF are shown at Figure 2.

### Recurrence of Atrial Fibrillation-Related Ischemic Stroke

Univariable analysis showed that AF-related ischemic stroke recurrence was associated with female sex, presence of dyslipidemia, a higher CHA2DS2-VASC score, a lesion pattern consisting of confluent plus additional lesions (compared with corticosubcortical lesions), and persistent AF. Multivariable analysis showed that persistent AF (HR = 2.94, 95% CI 1.69–5.26; *p* < 0.001) was the only factor independently associated with AF-related stroke recurrence (Table 2).

**Table 2:** Univariable and multivariable analyses of factors predictive of AF-related stroke recurrence.

	**AF-related recurrent ischemic stroke**			
	**Univariate**		**Multivariable**	
	**HR (95% CI)**	***p***	**HR (95% CI)**	***p***
Age	1.02 (1.00–1.04)	0.126		
Male	0.59 (0.37–0.94)	0.027	0.86 (0.47–1.58)	0.633
Hypertension	1.13 (0.68–1.88)	0.625		
Diabetes	1.57 (0.98–2.53)	0.063		
Dyslipidemia	1.72 (1.06–2.79)	0.028	1.30 (0.78–2.17)	0.312
CAD	1.74 (0.99–3.05)	0.055		
Smoking	1.02 (0.54–1.94)	0.947		
CHF	2.39 (0.97–5.90)	0.060		
CHA_2_DS_2_-VASC score	1.37 (1.13–1.67)	0.002	1.27 (1.00–1.61)	0.052
Type of AF				
Persistent	1			
Paroxysmal	0.29 (0.18–0.49)	<0.001	0.34 (0.19–0.59)	<0.001
AF diagnosis				
Before index stroke	1			
After index stroke	0.64 (0.40–1.02)	0.061		
Lesion pattern				
Corticosubcortical	1		1	
Cortical	1.59 (0.64–3.92)	0.316	1.37 (0.55–3.44)	0.503
Subcortical > 15 mm	2.00 (0.77–5.16)	0.154	1.94 (0.75–4.98)	0.171
Subcortical <15 mm	1.09 (0.30–3.93)	0.898	1.11 (0.31–4.00)	0.874
Small scattered	1.90 (0.79–4.58)	0.154	1.93 (0.80–4.66)	0.143
Confluent + additional	2.48 (1.20–5.13)	0.014	1.82 (0.88–3.75)	0.105
Multiple vascular territory	1.79 (0.81–3.93)	0.149	1.59 (0.73–3.47)	0.247
Carotid stenosis	0.60 (0.33–1.12)	0.111		
Mechanism of index stroke				
CE only	1			
Two or more	0.55 (0.25–1.20)	0.133		
Medication at discharge				
None	1			
Antiplatelet agents	1.07 (0.61–1.86)	0.823		
Anticoagulants	0.93 (0.29–3.01)	0.910		

*Results are presented as hazard ratios and (95% confidence intervals).*

*AF, atrial fibrillation; CAD, coronary artery disease; CHF, congestive heart failure; CE, cardioembolism*.

### Recurrence of Atrial Fibrillation-Unrelated Ischemic Stroke

Univariable analysis showed that AF-unrelated stroke was associated with paroxysmal AF, a pattern of small scattered lesions in a single vascular territory (compared with corticosubcortical lesions), and treatment with antiplatelet agents at discharge. The occurrence of AF-unrelated stroke was also associated with AF diagnosed before stroke. Multivariable analysis showed that paroxysmal AF (HR = 3.76, 95% CI 1.56–9.04; *p* = 0.003), AF diagnosed before index stroke (HR = 2.38, 95% CI 1.19–4.55; *p* = 0.014), small scattered lesions in a single vascular territory (HR = 3.19, 95% CI 1.18–8.63; *p* = 0.022 compared with corticosubcortical lesions), and treatment with antiplatelet agents at discharge (HR = 2.11, 95% CI 1.11–4.03; *p* = 0.024) were independently associated with AF-unrelated stroke (Table 3).

**Table 3:** Univariable and multivariable analyses of factors predictive of AF-unrelated stroke recurrence.

	**AF-unrelated recurrent ischemic stroke**			
	**Univariate**		**Multivariable**	
	**HR (95% CI)**	***p***	**HR (95% CI)**	***p***
Age	1.00 (0.97–1.02)	0.720		
Male	0.66 (0.35–1.24)	0.197		
Hypertension	0.96 (0.50–1.86)	0.912		
Diabetes	0.97 (0.48–1.99)	0.938		
Dyslipidemia	1.06 (0.51–2.23)	0.876		
CAD	0.96 (0.38–2.44)	0.926		
Smoking	0.47 (0.15–1.55)	0.216		
CHF	0.85 (0.12–6.10)	0.867		
CHA_2_DS_2_-VASC score	1.02 (0.81–1.29)	0.845		
Type of AF				
Persistent	1		1	
Paroxysmal	4.14 (1.74–9.86)	0.001	3.76 (1.56–9.04)	0.003
AF diagnosis				
Before index stroke	1			
After index stroke	0.42 (0.21–0.83)	0.013	0.42 (0.22–0.84)	0.014
Lesion pattern				
Corticosubcortical	1		1	
Cortical	1.57 (0.51–4.83)	0.433	2 (0.65–6.2)	0.228
Subcortical > 15 mm	0.88 (0.19–4.15)	0.874	1.02 (0.23–4.62)	0.980
Subcortical <15 mm	1.72 (0.45–6.58)	0.431	1.94 (0.51–7.48)	0.333
Small scattered	2.94 (1.10–7.88)	0.032	3.19 (1.18–8.63)	0.022
Confluent + additional	0.97 (0.31–3.06)	0.960	1.61 (0.5–5.14)	0.422
Multiple vascular territory	1.79 (0.67–4.80)	0.249	2 (0.74–5.42)	0.171
Carotid stenosis	0.88 (0.42–1.85)	0.741		
Mechanism of index stroke				
CE only	1			
Two or more	1.09 (0.48–2.47)	0.831		
Medication at discharge				
None	1			
Antiplatelet agents	2.52 (1.34–4.76)	0.005	2.11 (1.11–4.03)	0.024
Anticoagulants	0.72 (0.01–5.19)	0.740	0.58 (0.08–4.13)	0.585

*Results are presented as hazard ratios and (95% confidence intervals).*

*AF, atrial fibrillation; CAD, coronary artery disease; CHF, congestive heart failure; CE, cardioembolism*.

## Discussion

This study found that AF diagnosed before the index stroke and the lesion patterns were independently associated with recurrence of ischemic stroke. Two-thirds of ischemic stroke recurrences were AF-related, whereas the remaining one-third was regarded as AF-unrelated. Regarding the type of stroke recurrence, persistent AF was associated with AF-related stroke recurrence, whereas paroxysmal AF was associated with AF-unrelated stroke recurrence.

AF is frequently detected by cardiac monitoring during patient admission during the acute stage (11). It is unclear whether AF observed in this stage is the cause of stroke or is triggered by the stroke (neurogenic AF) (12). A previous study found that the incidence of stroke recurrence was low in patients with AF detected after stroke, suggesting that their pathophysiology may differ (13). Similarly, the present study showed that AF diagnosed before stroke was more frequent in patients with ischemic stroke recurrence than those without. The influence of AF detected after stroke due to an autonomic or inflammatory response may differ from that of longstanding AF diagnosed before stroke (14). However, it did not differ according to the type of stroke recurrence.

The present study found that the type of AF was associated with the type of stroke recurrence. Although the rate of early ischemic recurrence was found to be higher in patients with persistent than with paroxysmal AF, this difference was no longer observed after adjustment for relevant risk factors (15). However, *post-hoc* analysis of the major non-vitamin K-dependent oral anticoagulant trials showed that the rates of thromboembolic events and deaths were lower in patients with paroxysmal than with persistent AF (16). In the present study, which assessed ischemic stroke recurrence according to stroke mechanism, persistent AF was associated with AF-related ischemic stroke recurrence. On the other hand, paroxysmal AF was relatively more associated with AF-unrelated ischemic stroke recurrence, which may be explained by the chance that paroxysmal AF may not be directly be the cause of AF-unrelated ischemic stroke recurrence.

Single small subcortical lesions are common in small vessel disease, and small scattered lesions in a single vascular territory are common in ischemic stroke caused by a corresponding artery stenosis (9). Although the type of index stroke and the presence of concomitant carotid stenosis were not associated with AF-unrelated stroke recurrence, lesion patterns representing embolism from a concomitant vascular stenosis were associated with AF-unrelated stroke recurrence. Non-stenotic atherosclerotic plaques may have contributed to ischemic lesions in a single vascular territory (17). Interestingly, the conventional CHA2DS2-VASC score was not significantly associated with AF-unrelated or AF-related stroke. Because factors associated with AF itself and imaging characteristics showed greater associations with AF-related or AF-unrelated stroke, a prediction model including these factors may increase its ability to predict each type of stroke recurrence.

The present study had several limitations stemming from its prospectively registered observational study design. First, our data are prone to a possible residual bias with methodological shortcomings. Several factors important for stroke recur, such as the presence of peripheral artery disease or the reason for not receiving anti-thrombotics, were not obtained. Second, though we have divided recurrent stroke to AF-related and unrelated strokes, there may be some controversies. However, DWI lesion pattern well-reflects the stroke mechanism, and the stroke mechanism was determined by local investigators and was once more confirmed centrally. Third, because this study was a non-randomized comparison of an observational registry, the relationship between treatment and outcome may have been influenced by measured or unmeasured confounding factors. For example, although treatment with antiplatelet agents at discharge was associated with AF-unrelated stroke recurrence, patients who were at risk of a non-CE recurrence may have received antiplatelet agents.

Despite these limitations, our study showed that the time of AF diagnosis was associated with stroke recurrence and that the type of AF affected the type of stroke recurrence among ischemic stroke patients with AF. AF diagnosed before the index stroke increases the risk of stroke recurrence. Persistent AF was more associated with AF-related stroke recurrence, whereas paroxysmal AF was more associated with AF-unrelated stroke. Ischemic lesions in a single vascular territory predicted the recurrence of AF-unrelated stroke.

## Data Availability Statement

The raw data supporting the conclusions of this article will be made available by the authors, without undue reservation.

## Ethics Statement

The studies involving human participants were reviewed and approved by Kyung Hee University Hospital. Written informed consent for participation was not required for this study in accordance with the national legislation and the institutional requirements.

## Author Contributions

BK contributed to the conception and design of the work, the acquisition, analysis and interpretation of data, and drafting of the content. Y-HH, M-SP, J-TK, K-HC, J-MJ, SY, CK, KO, T-JS, Y-JK, K-YP, J-MK, J-HP, JC, J-WC, OB, and G-MK contributed to the analysis of the work and critically revised the content. SH contributed to the acquisition and analysis of the data and critically revised the content. W-KS contributed to the conception and design of the work, the acquisition, analysis and interpretation of data, and critically revision of the content. All authors contributed to the article and approved the submitted version.

## Funding

This study was supported by a grant from the Korean Neurological Association (KNA-17-MI-10: W-KS).

## Conflict of Interest

The authors declare that the research was conducted in the absence of any commercial or financial relationships that could be construed as a potential conflict of interest.

## Publisher's Note

All claims expressed in this article are solely those of the authors and do not necessarily represent those of their affiliated organizations, or those of the publisher, the editors and the reviewers. Any product that may be evaluated in this article, or claim that may be made by its manufacturer, is not guaranteed or endorsed by the publisher.
